# State of the Art of Hydrogel Wound Dressings Developed by Ionizing Radiation

**DOI:** 10.3390/gels9010055

**Published:** 2023-01-10

**Authors:** Maria Demeter, Anca Scărișoreanu, Ion Călina

**Affiliations:** National Institute for Lasers Plasma and Radiation Physics, 409 Atomiștilor, 077125 Măgurele, Romania

**Keywords:** hydrogel, ionizing radiation, wound dressings, crosslinking, healing

## Abstract

The development of an ideal hydrogel wound dressing with excellent characteristics is currently a significant demand in wound therapy. The ideal hydrogel wound dressing must provide a moist environment between the wound and the dressing, promote wound healing, absorb excess exudate and toxins, be completely sterile, and not adhere to the wound. The evolution and current status of research on hydrogel wound dressings obtained exclusively through production by ionizing radiation are discussed in this paper review, along with the preparation methods, properties, standard characterization techniques, and their applications in wound dressing. First, we described the methods for synthesizing hydrogel wound dressings with ionizing radiation. Then, standard methods of characterization of hydrogel wound dressings such as gel fraction, swelling degree, sol–gel analysis, rheological properties, morphology, moisture retention capability, and water vapor transmission rate have been investigated. In the end, specific attention was paid to the drug release, antibacterial performance, and cytotoxicity of hydrogels. Moreover, the application of hydrogel in regenerative medicine as wound healing dressing was covered.

## 1. Introduction

The largest organ of the human body is the skin, which represents the main defence barrier against various pathogens commonly encountered in the environment. Wounds on the skin break these barriers and form entry gates for external pathogens [1]. To overcome the exposure of the wounds to the external environment, it is recommended to immediately cover them with suitable dressings depending on the severity of the wound. Dry dressings generally used in medical practice provide only physical protection, they do not help the healing of the wound.

In recent decades, hydrogels based on natural/synthetic polymers have become a modern dressing alternative for wound care, due to the excellent properties of polymers that absorb a large volume of water through the hydrophilic functional groups. Hydrogels derived from polymers can be obtained through several methods that ensure the polymer’s crosslinking. Physical crosslinking can occur through ionic interactions [2], hydrogen bonds [3], crystallization [4], hydrophobic interaction [5], or protein interaction [6]. Chemical crosslinking methods include free radical polymerization [7] and enzymatic reaction [8]. Since physical methods do not ensure homogeneous crosslinking in the hydrogel structure, and in the case of chemical crosslinking, the inclusion of crosslinking agents leads to a decrease in the biocompatibility of the hydrogel, the best crosslinking method that removes the disadvantages of the methods above mentioned is radiation crosslinking. 

The ionizing radiation technology applied to hydrogel synthesis was first reported in 1958 when γ-radiation was used for PVA crosslinking [9]. In this period, the first studies on the crosslinking of poly(vinyl alcohol) (PVA) and poly(N-vinyl pyrrolidone) (PVP) by irradiating aqueous solutions with γ-radiation were also achieved [10,11]. Charlesby and Alexander were the first researchers who reported the crosslinking of PVP with ionizing radiation. Over the years, various other water-soluble polymers have been treated or crosslinked with ionizing radiation, and this treatment is often also a step in the preparation of new polymeric biomaterials. Thus, the radiation crosslinking technique has become one of the standard methods for obtaining hydrogels [12]. 

In the following decades, the study of hydrogels remained focused on networks with a relatively simple structure obtained by chemical crosslinking of synthetic polymers, with applications in medicine (ophthalmology and controlled drug release). Hydrogels with a simple network structure offered the possibility of characterizing some of their physicochemical properties, such as crosslinking density and the elucidation of diffusion processes.

The synthesis of hydrogels for biomedical applications by irradiation with ionizing radiation has seen constant development since the late 1960s, as mentioned in the papers and patents published by Sakurada and Ikada [13], Kaetsu [14,15], and Hoffman [16]. 

A substantial contribution in this field was made by Professor Janusz M. Rosiak and his collaborators from the Technical University of Lodz, Poland, the main inventor of the technology for obtaining hydrogel dressings by irradiation. In the 1980s, Rosiak et al. obtained PAAM hydrogels by crosslinking with γ-radiation. The obtained hydrogel was intended to be used as a dressing for skin drug release [17]. 

Later, in 1989, Janusz M. Rosiak and his collaborators patented the first commercial hydrogel for biomedical applications based on PVP known under the trade name (Aqua-Gel^®^), currently marketed in Poland and some Central European countries. This hydrogel has bacterial barrier properties eliminating wound superinfection, inhibiting the loss of body fluids, allowing oxygen access to the wound, and, in general, accelerating the healing process. The hydrogels developed by Rosiak consist of water, poly(N-vinyl-2-pyrrolidone) (PVP), poly(ethylene glycol) (PEG), and agar. In the end-use form, they are supplied as transparent sheets with a thickness of a few millimeters, containing over 90% of water [18].

Another aspect related to the production of hydrogels using irradiation as the main production technology is that recently, worldwide, natural and synthetic polymers that are soluble in water, biodegradable, and biocompatible are used more and more, to the detriment of synthetic ones derived from petroleum. The second aspect is related to the promotion of non-polluting technologies and processing with ionizing radiation, especially because irradiation with electron accelerators is a non-polluting technology. Due to the diverse and numerous applications, the number of scientific studies on the preparation of hydrogels by irradiation with ionizing radiation has increased rapidly, especially after the year 2000.

Since 2003, at the Institute of Energy and Nuclear Research (IPEN) in Brazil, there has been a fully automatic production line for the production of dressings in the form of hydrogels commercially named Biogel^®^. Important contributions were made by Benamer, Darwis et al., who reported obtaining pure and stable PVP hydrogels using γ-radiation, at concentrations of 7–10% PVP and doses of 2–3 kGy, in saturated solutions in the environment of inert Ar or N_2_ [19,20]. Can et al. studied the effect of γ-irradiation on aqueous solutions of PVP in the presence of persulfate anions [21]. Ozurek, Dafader et al. studied hydrogels based on PVP/tartaric acid and agar [22,23]. Dergunov [24,25], Sohail [26], and Abd Alla [27] studied hydrogels sensitive to pH variations obtained from PVP/chitosan or PVP/AAc. Gottlieb [28] studied hydrogels obtained from PVP and PVME poly(vinyl methyl ether) sensitive to temperature variations used to obtain controlled drug release systems. Awadallah [29] studied hydrogels obtained by γ-irradiation from PVA/PVP/PAA, used in meniscus replacement treatment.

The most important PVP-based hydrogels obtained using γ-radiation crosslinking for biomedical applications (dressings, controlled drug release systems) were made by Razzak et al. [30]. By adding PVA, they obtained a hydrogel with high resistance to *E. coli*. Himly et al., by adding PEG, obtained elastic, transparent, flexible hydrogels, impermeable to bacteria, which absorb a large amount of water and are easy to remove [31]. Lugao et al., by irradiation of aqueous solutions of PVP (6%) and PEO (3%), obtained hydrogels with increased elasticity [32]. 

Pekel et al. synthesized biodegradable hydroxypropylmethylcellulose (HPMC) in a paste-like condition using e-beam irradiation and demonstrated that the HPMC with a higher degree of substitution gave a higher gel content at each concentration and irradiation dose above 20 kGy [33]. Rashid et al. evaluated the effect of γ-irradiation on chitosan (CS) samples. They showed that the degree of deacetylation, swelling degree, and antimicrobial activity of CS improved after irradiation [34]. 

Wang et al., by adding CMC, obtained hydrogels with better mechanical strength than that of pure CMC hydrogel, and with better flexibility and equilibrium swelling than that of pure PVP hydrogel. These hydrogels show a similar moisture retention capacity to that of a commercial hydrogel-based dressing [35]. Park and Nho, by adding Aloe Vera to the hydrogel obtained from PVP and PVA, obtained dressings with superior qualities [36]. Nho and Park, by adding chitosan to the hydrogel obtained from PVP and PVA, manufactured dressings with superior qualities for rapid healing and stopping bleeding [37]. Singh et al., by adding alginate and Ag nanoparticles, obtained a hydrogel with a strong antimicrobial effect [38]. Song et al. fabricated a GEL/alginate/CMC hydrogel by radiation-induced crosslinking with good biocompatibility and accelerated wound healing [39]. El-Rehim et al., by adding PAA, obtained a hydrogel used as a polymer matrix for the release of drugs in the gastric environment where the solubility of PVP is extremely low [40]. Kadlubowski et al., by adding PAA, obtained a hydrogel very sensitive to pH variations [41].

Until now, most of the scientific works related to hydrogels manufactured to be used as wound dressings present mostly studies, which are based either on the development of new innovative products, with multiple functionalities, or are focused on the characterization of newly developed materials, using methods of very complex analysis. Other studies are also focused on the detailed description of the materials used for the manufacture of hydrogels and their properties.

However, we have identified that in the specialized literature, there is a need for a new perspective on the properties a hydrogel must fulfill to be used successfully in wound dressing applications. The current review aims to include the physicochemical and biological properties required for hydrogel dressings for wound healing.

In general, a hydrogel designed for wound dressings must demonstrate a series of physicochemical, biological, and mechanical properties: be insoluble (gel fraction properties), have the power to absorb liquids (be absorbent), be physically and chemically stable in different pH environments, be non-toxic, be biocompatible, biodegradable, be elastic from a mechanical point of view, allow the transmission of gases, keep its moisture, not adhere to the skin, improve the rapid healing of wounds, and be cheap to manufacture [42]. 

**Figure 1:**
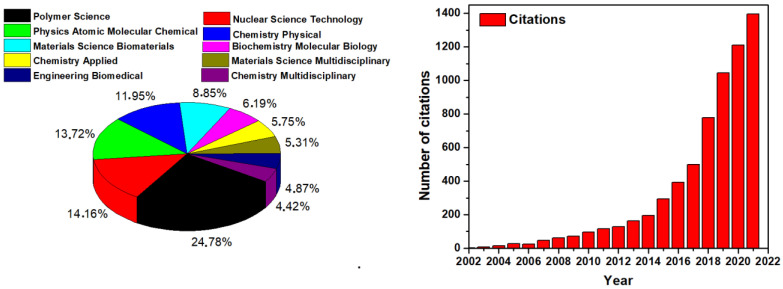
Papers published on hydrogel wound dressings prepared by ionizing radiation in various disciplines and the number of citations per year.

Until 2022, about 157 publications on hydrogel wound dressing obtained by ionizing radiation were published, with a total of 6144 citations in the last 20 years (Figure 1). The most relevant papers were published in polymer science topics as well as in nuclear science technology.

## 2. Wound Dressing Hydrogel Crosslinking Using Irradiation Technology

High-energy irradiation of aqueous solutions of polymers leads to the formation of radicals on the polymer chain resulting in the scission of the C–H bonds. In addition, the hydroxyl radicals resulting from the radiolysis of water molecules are attached to the polymer chains, forming macroradicals. The crosslinked structure of hydrogels is given by the recombination of macroradicals [43]. Using irradiation technology to crosslink natural/synthetic polymers, hydrogels based on poly(acrylic acid), poly(ethylene glycol), poly(vinyl alcohol), or poly(methyl vinyl ether), were obtained [44,45,46].

This crosslinking method has several advantages: it is carried out in aqueous solutions, at physiological pH and room temperature, does not require other special conditions, and the irradiated polymers change their physical and chemical properties at the same time during irradiation [47]. Moreover, no chemical initiators are required for hydrogel crosslinking [48]; this is beneficial for biomedical applications because the resulting medical devices are also sterilized during irradiation.

Internationally, we have identified several companies that produce dressings in the form of hydrogels obtained by ionizing radiation. Some of these are produced by e-beam crosslinking and marketed by the KikGel company (Ujazd, Poland), under various trade names: BurnTec^®^, HydroAid^®^, and Neoheal^®^, and their main use is the treatment of burns and various procedures in plastic surgery (http://kikgel.com.pl, accessed on 12 December 2022). Nexgel (Langhorne, PA, USA) produces hydrogel based on PEO, PEG, or PVP by crosslinking with e-beam for wound healing care, cosmetics, and other applications (https://nexgel.com, accessed on 12 December 2022). Alliqua Inc. (Langhorne, PA, USA) produces SilverSeal^®^ and Hydress^®^ products, which are hydrogels for wound dressings made of polymers crosslinked by e-beam and are used for superficial wounds, abrasions, burns, and dermal/leg ulcers (https://www.prnewswire.com/news-releases/alliqua-signs-distribution-agreement-for-silverseal-and-hydress-wound-dressings-228603251.html, accessed on 12 December 2022).

Besides the radiation simultaneously crosslinking and sterilizing in one step, easy control of hydrogel physical properties can be obtained by controlling the absorbed dose and polymer composition. The biomaterials, methods of preparation, and irradiation dose used to develop hydrogel wound dressings are summarized in Table 1.

## 3. Characterization of Hydrogel Wound Dressings Prepared by Ionizing Radiation

### 3.1. Gel Fraction and Swelling Degree

A mandatory step in the evaluation of hydrogel dressing produced by radiation crosslinking is the quantification of the hydrogel formation, in other words, the formation of insoluble material [115]. This step can be easily done using a classical gravimetrical method and can be performed as well using a Soxhlet extraction procedure [116]. 

The crosslinked hydrogel content (gel fraction), GF, is expressed as the mass of the insoluble gel sample in the dry state (m_d_) divided by the initial mass (m_i_) of the sample in the dry state, after immersion in water for a standardized period: 16 h [117], or 48 h [118].

One of the characteristic features of hydrogel is its swelling response when subjected to various environmental variations such as water, pH, and ionic strength. Hydrogels’ good absorption properties are the most important characteristics, especially when intended to be used for biomedical applications. The swelling of a hydrogel is measured in terms of the swelling ratio of swollen gel to dry gel. One of the most crucial physical aspects of hydrogels is their soluble nature. This can be determined by calculating the insoluble component of a dried sample following immersion in water for 48 h at room temperature.

The body’s blood contains about 90% water, and the excellent swelling properties of the hydrogels make the gel absorb a large amount of water in the blood favoring the bleeding to stop. Table 2 includes the values for gel fraction, swelling degree in water, and PBS of hydrogel wound dressing irradiated between 25–30 kGy.

### 3.2. Sol–Gel Analysis

For the hydrogels obtained by irradiation technology, sol–gel analysis is a useful tool to determine the insoluble gel and the radiochemical yield of the crosslinking. The radiochemical yield, (G) represents the number of units (molecules, atoms, ions) transformed by ionizing radiation following the absorption of a quantity of radiant energy of 100 eV and is expressed in mol·J^−1^ [121]. The sol–gel analysis method is applied in the case of polymer mixtures above the gelation dose and can estimate also the scission yield. The radiochemical yield of scission under standard irradiation conditions was found to be close to zero for most non-ionic polymers such as PVP, PVA, or PAAM [122]. The main parameters specific to irradiation technology are determined with the Charlesby–Rosiak equation, which is the basis of the numerical analyses corresponding to the gel/absorbed dose curves which are integrated into a calculation software called GelSol95 (made at Lodz University of Technology, Division of Applied Radiation Chemistry, Poland) [123]. The sol–gel parameters determined below using GelSol95 are the degradation/crosslinking ratio (p₀/q₀), the gelation dose (Dg), and the virtual dose (Dv). When the (p₀/q₀) is less than 2, for polymer mixtures that form insoluble fractions after irradiation, it can be said that crosslinking prevails more than degradation [124].

To determine the changes that take place in polymer solutions after irradiation with ionizing radiation, first are determined the parameters: radiochemical yield of the crosslinking G(X) and the radiochemical yield of the scission G(S), which give information about the number of new bonds formed per unit of absorbed energy, and the number of broken chains, respectively. The two parameters change simultaneously, and the final result of the irradiation is given by the value of their yields [125]. Considering that the unit of absorbed dose is Gy, this corresponds to 1 Joule per 1 kg of irradiated material. For the crosslinking process, the most important is the radiochemical yields of the radiolysis products of water (HO**∙**, H**∙**, $e^{-}{}_{\mathrm{aq}.}$, H_2_O_2_, H_2_, and H^+^), which are very well established (2.8, 0.6, 2.7, 0.7, 0.5, and 2.7 × 10^−7^ mol J^−1^, respectively). The radicals HO**∙** and H·, formed in the process of radiolysis of water, are the main species responsible for the formation of macroradicals. The HO**∙** radical extracts a hydrogen atom, and forms a water molecule, and the hydrogen radical extracts a hydrogen atom from the polymer molecule, resulting in an H_2_ molecule, which accumulates in the form of gas bubbles during the gelation process. When a polymer–water system is exposed to radiation, a series of macroradicals are formed, which participate in recombination reactions [126].

To determine the crosslinking parameters specific to collagen and PVP mixtures, Demeter et al. used sol–gel analysis. They observed that in some mixtures the gelation started before the chosen irradiation dose, depending on the amount of collagen in the mixture [127]. Depending on the composition of the six analyzed hydrogels, the ratio was between 0.09–0.79, which indicates that the crosslinking process prevailed and the degradation is negligible. The same thing is observed in the case of the values obtained for G(X) and G(S), i.e., G(X) > G(S), which means that the polymers were predominantly crosslinked [119]. In another study, the sol–gel analysis method was used for PAAM hydrogels in which poly (L-ascorbic acid) was incorporated. It was observed that the values of the degradation/crosslinking ratios ranged between 0.03 and 0.30 and predominantly indicated the crosslinking of polymer chains compared to degradation. They also have higher values when the content of poly (L-ascorbic acid) increases in the polymer mixture [128].

In the case of quaternary hydrogels from CS, PVP, PEG, and PAA obtained by e-beam irradiation, the ratio (p₀/q₀) had a value of 0.28. Additionally, the values for G(X) and G(S) when the hydrogel was irradiated with 25 kGy were equal to 0.23 µmol/J and 0.13 µmol/J, respectively, which suggests that the crosslinking yield was higher than the degradation yield. For the other hydrogel (made of CS, PVP, and PEO), the (p₀/q₀) ratio was 0.51, which means that the hydrogel showed polymer chain scissions in a moderate proportion and the values for G(X) and G(S) after irradiation were equal to 0.22 µmol/J and 0.25 µmol/J respectively, which means that the two yields are almost equal G(X) ≈ G(S) [52]. G(S) was determined for CS in solid state and aqueous solution after being irradiated with γ-irradiation and subjected to sonochemical degradation treatment [129]. The values obtained were equal to 0.6 µmol/J for CS in solid state and 0.19 µmol/J for CS in aqueous solution, values close to those reported in other studies. The crosslinking yield has lower values for polymers in the solid state than in the aqueous solution, due to reactions of transient species resulting from water irradiation [130]. The gelatin (GEL) hydrogels were crosslinked at 30 kGy and showed that the radiochemical yield ratios and Dg increased with the initial concentration of GEL [75]. The sol–gel analysis is a useful and necessary method for determining the characteristics of polymeric hydrogels to understand their behavior when used as biomedical devices.

### 3.3. Rheology and Mechanical Properties

The elasticity or stiffness properties of a hydrogel can be highlighted by a series of mechanical parameters [131]. The most used techniques for the quantitative description of the flow resistance (viscosity) and the deformation resistance of polymer solutions are rheology/viscometry. Rheology is a technique that provides valuable information both on the molecular structure (determining the size of the macromolecular chains and the crosslinking density), on the behavior of the hydrogel, as well as on the self-assembly mechanisms.

For polymer hydrogels, rheology is probably the most important physicochemical characterization technique [132]. When a hydrogel sample is inserted between two horizontal disks (or cylinders), and after applying an oscillating stress (or force) of a certain value to one of the disks, the motion induced in the other disk is decomposed into two components (in-phase and out-of-phase). The storage modulus (G′, elastic component) and loss modulus (G″, viscous component) can be measured as a function of applied stress or oscillation frequency. In general, to identify the formation of a hydrogel by rheology two experiments are commonly used: the linear response of the modules (G′ and G″) to a fixed stress of small amplitude applied by the frequency variation, and the second one refers to the nonlinear response of the modules at a fixed frequency by changing the shear force. For a typical hydrogel, G′ should be invariant with frequency up to yield stress corresponding to the transition from the gel state to the ground state and should exceed G″ by at least an order of magnitude. If the G′ decreases rapidly by applying a force, this indicates the breaking of the macromolecular network. The behavior and magnitude of the G′ and G″, as well as the flow stresses depending on the applied force can be mathematically modeled and lead to conclusions regarding the gel structure [133]. The values of the G′ and G″ moduli can be related to the crosslinking density (Ve) by using the rubber elasticity theory. In the reference [134], the equation for crosslinking density determination for hydrogels produced by radiation synthesis is described very well. Concerning the rheological demands for ideal wound dressing, the hydrogel must have adequate stretch and compression properties, an elastic modulus comparable to human skin, and good adhesion, but not excessively adhesive, to support deformation [135]. Similarly, in further paragraphs, we emphasize the rheological and mechanical properties of hydrogel wound dressings produced at the legally accepted sterilization dose.

Dynamic mechanical analysis performed on gels fabricated of PHEMA and P(HEMA/Itaconic Acid) showed improvement in the mechanical strength of the polymeric network if the itaconic acid content is not higher than 3.5 mol%. The moduli values were determined in the relaxation state, meaning after γ-irradiation crosslinking was between 4.73–18.4 kPa at 25 kGy [81]. PVP-k-carrageenan hydrogels wound dressings with the composition based on 10% *w*/*w* PVP, 2% *w*/*w* KC, 0.3% *w*/*w* KCl, and 1.5% *w*/*w* PEG showed a fracture strength of 1750 kPa and a toughness 83.6 mJ for the 25 kGy sample by comparison with irradiated ones. As the authors described, these results confirm the influence of radiation action on the crosslinking density of hydrogels. The hydrogels in question, besides their mechanical properties, exhibit fluid absorbency, elasticity, and transparency, and are easy to remove [104]. PVA-agar-carrageenan presented a drastic increase in elongation and tensile strength by adding 1% agar. These dressings were synthesized using γ-irradiation in a single irradiation stage, so the gel formation and sterilization have been performed at 25–30 kGy [82]. Lugão et al. have investigated the rheological properties of PVP hydrogels and the influence of some additives such as PEG, PEO, and glycerol on the gel properties using γ-irradiation synthesis. The hydrogel formulation containing PVP 6% and PEO 3% showed an elastic modulus of 33,000 Pa and the formation of a transparent fluid gel with high elasticity [32]. The PVA/cellulose acetate/gelatin hydrogels composites crosslinked by γ-irradiation at 25 kGy and loaded with 20 mM AgNO_3_ showed an improved tensile strength of 34,000 Pa [85]. The nanosilver/GEL/CM-chitosan hydrogels possessed interconnected porous structures and had a compressive modulus of 54,000 Pa at 1 mM AgNO_3_, which slightly reduced with the increase of nanoparticles. The highest compressive strength was obtained at 10 mM AgNO_3_. The hydrogels were synthesized using the radiation-induced reduction of silver ions and crosslinking in the same fabrication step [62]. The CS, GEL, PVA, and polyacrylamide sterile hydrogel wound dressings produced by γ-irradiation at 45 kGy showed a tensile strength of 6.68 MPa and 2.45 MPa. The results indicated that hydrogel films can be stretched, folded, and are resilient; moreover, these dressings can provide mechanical protection to the wound and can mimic artificial skin function [136].

PVA/CM-CS/honey hydrogels were prepared by irradiation at 25–40 kGy and by freeze-thawing; this approach allowed for obtaining dressings with higher mechanical strength [86]. Hydrogel wound dressings prepared from PVP, PEG, and agar showed constant values of stress at break within 25–35 kGy of about ~4000 Pa and much higher at 50 kGy due to increased crosslinking density. The higher radiation dose decreased the strain at break [106]. E-beam synthesis of PVP/PEG and PVP/PEG/starch wound dressings have been performed also at the legal sterilization dose of 25 kGy. The PVP content increased the tensile strength above 25 MPa, while the PEG content increased the elongation at break. The irradiation dose has a defining role in the mechanical properties, mainly by increasing them [60]. Hydrogels based on polyglutamic acid and GEL, showed at 25 kGy a strong tensile strength of 1 MPa and an elongation at break of 377%. Higher radiation doses of 40 kGy produced an excess of crosslinking and led to a fragile and brittle hydrogel [74]. Quaternary ammonium CS/PVA/PEO hydrogels prepared using γ-irradiation reached a tensile strength of ~34 MPa at 40 kGy. This study proves that a higher irradiation dose induced radiation degradation effects causing a decrease in tensile strength, while a higher content of quaternary ammonium chitosan decreased the elongation [76]. The tensile strength of 2.2 MPa was obtained for hydrogel wound dressings of CS/GEL/PVA crosslinked by γ-irradiation at 40 kGy. Their mechanical properties were mainly influenced by the polymer’s weight ratios [69]. PVP/PEG/agar hydrogel wound dressings produced at pilot scale using γ-irradiation crosslinking and sterilization demonstrated the same quality from batch to batch. Following the compression tests, a slow decrease of the compression rate of 20% until 7 h and about 70% at 24 h was observed [105]. The PVA/PVP/CS hydrogels crosslinked at 25 kGy presented a tensile strength between 0.38 to 0.72 MPa and an elongation at the break between 217% to 277%. The same hydrogels were treated by the freeze-thaw procedure, which suggested that a higher dose of 25 kGy affects the mechanical properties, decreasing the strength of hydrogel at 0.37 MPa and elongation at 178% [97]. The hydrogels obtained from PVA and PVP at 20 kGy presented a very low tensile strength of 147 Pa and an elongation of 175%. Advanced decreasing of mechanical properties was found after irradiation above 20 kGy [92]. PVA/ws-CS hydrogels made using γ-irradiation (30 kGy) exhibit almost no temperature dependence due to the stable chemical crosslinking structure. By adding 1% chitosan, the elastic modulus (G′) was found to be ~5000 Pa [100]. Storage (G′) and loss (G″) moduli of e-beam crosslinked agarose hydrogels corresponded to 12.8 kPa. After e-beam irradiation with 30 kGy, the viscoelastic properties underwent a reduction of more than 20% compared to the initial moduli [49]. Thermally resistant hydrogels were made from PVA by e-beam irradiation and PVA acetalization followed by additional grafting of AA on the final dressing. The hydrogel gained the maximum tensile strength of 20 MPa at a very high radiation dose of 100 kGy. After steam autoclave sterilization, the tensile strength decreased to 10 MPa and the elongation at break increased to 300%. The hydrogel adhered to the burning surface and healed the burns faster compared with a typical dressing [54]. The hydrogel wound dressings formed by radiation-induced crosslinking using moringa oleifera gum/carbomer were developed to be used as a drug carrier for levofloxacin release. The optimal γ-irradiation dose was set at 13.68 kGy. The principal mechanical properties were tensile strength = 2.68 MPa, elongation at break = 64.49%, burst strength = 3.66 N, stress–relaxation = 47.10, percentage resilience = 28.25, and folding endurance = 234. The hydrogel dressings were hemocompatible, antioxidant, and bioadhesive [78]. CS/PVA hydrogels crosslinked using γ-irradiation showed a tensile strength of ~0.4–0.5 MPa at 20–50 kGy. In these conditions, the elongation at break showed a systematic decrease while increasing the PVA content, as well as with increasing the irradiation dose [87]. Antibacterial AgNP/GEL/PVA hydrogels were produced using γ-irradiation at 30 kGy using a weight ratio of gelatin: PVA of 60:40. The higher AgNP content revealed a negative impact on the mechanical properties of these hydrogels. At 0.25% AgNP content, the hydrogels presented a tensile strength of 0.0256 MPa and an elongation percentage of ~180% [63]. PVA/alginate hydrogels were produced using γ-irradiation. The tensile strength of the PVA/alginate hydrogels increased as the concentration of alginate was lower and as the radiation dose increased. The hydrogel swollen samples showed a tensile strength of 14 MPa and an elongation of over 70% [84]. Hydrogels that exhibit antibiotic activity containing PVA and cetylpyridinium chloride were developed using γ-radiation synthesis. Their mechanical properties were assessed using dynamic viscoelastic measurements. The results indicated a G′ value of 10,500 Pa, this value being affected by increasing the antibiotic active ingredient. The authors observed that hydrogels prepared only from PVA had a G′ of 7000 Pa, and were very soft and strong adhesives [96]. Hydrogels based on PEO and PVA containing high PVA content (20% and 30%) were produced by e-beam synthesis at 60 kGy and showed high strength of 0.2 to 0.35 MPa and elasticity of 500%. The tensile strength and elongation decreased with increasing doses because of the increase in crosslinking [53]. Collagen-PVP superabsorbent hydrogels were obtained using e-beam irradiation. The hydrogels prepared without crosslinking agents showed very low elastic moduli, below 66.5 Pa; in comparison, the hydrogels prepared with 0.5% crosslinking agent and irradiated showed maximum elastic modulus of 14,000 Pa. Higher irradiation dose above 25 kGy, decreased the G′ value considerably [119]. Gelatin-PVP hydrogels functionalized with gallic acid were developed to obtain hydrogels with antioxidant properties. The microindentation assay demonstrates the formation of hydrogels with an elastic modulus between ~15,800 to ~44,800 Pa. A dose of 15 kGy was selected to obtain mechanically stable materials having adequate stability at manipulation [75]. PVP/kappa-carrageenan/PEG hydrogel dressing produced at 25 kGy using γ-irradiation showed a higher tensile strength of 0.05 MPa than commercial hydrogels based on PVP and PVA (0.04 MPa). The elongation at a break of 138% was almost similar to that of PVP commercial hydrogels (142%) [103]. PVP/PEG hydrogel membranes reinforced with MMA and PP fibers were prepared using e-beam irradiation at 20 kGy. The tensile strength and elongation depended on the grafting degree and varied between 0.02 and 0.16 MPa and ~50–70%, respectively [56]. Hydrogel dressings based on PVA, NVLC, and HEA presented at 25 kGy a tensile strength of 547 Pa, elongation at break of 115%, and elastic modulus of 3.6 kPa. Elastic modulus and tensile strength increased as a consequence of sterilization dose, while elongation decreased [137]. A sterile hydrogel film made from 3.75% tragacanth gum, 3.125% sodium alginate, and 5% PVA was obtained at 18.2 kGy using γ-irradiation. The films showed a mechanical strength of 9.56 N, which classified them as adequate for wound dressing applications [113]. The same authors reported the fabrication of hydrogels containing 2.5% tragacanth gum, 4% SA, and 1.12 × 10^−2^ mol/L Aam at a total dose of 34.7 kGy. These hydrogels were characterized by a tensile strenght of 2.45 MPa and elongation of 40.15% [112]. The e-beam crosslinking of the collagen gels leads to a strong increase in G′ modulus up to 69.3 kPa at 100 kGy. At 25 kGy, the collagen hydrogel had a G′ of 6174 Pa. Higher irradiation doses induce the formation of a compact network that restricts the movement of collagen chains and prevents water from being absorbed [51]. Antibacterial composite hydrogels prepared from CS/PVA/TA at 30 kGy using γ-irradiation have a tensile strength of 200 kPa and 424% elongation at break. The mechanical properties varied as a function of the CS molecular weight [70]. Bacterial cellulose (BC)/AA hydrogel wound dressing was synthesized using e-beam irradiation at 35 and 50 kGy. Hydrogels fabricated with 40% AA and 60% BC showed a tensile strength of ~ 1.5 MPa and elongation of about 300% [50]. The e-beam synthesis of PVP/PEG/agar wound dressing at 20 and 25 kGy produced a decrease in tensile strength and elongation with increasing irradiation dose. The tensile strenght and elongation varied from 17 to 27 kPa and 120–160%, respectively. These parameters were strongly influenced also by the %PVP and %PEG, as well as by the PVP molecular weight [58]. The PVA/GEL bilayer hydrogel achieving both wound dressing and antiadhesion capabilities was developed using γ-irradiation. At the dose of 30 kGy, the PVA side showed a modulus of elasticity of about ~12 kPa, and the other side with GEL had ~14 kPa [138].

### 3.4. Network Parameters

To tailor the molecular weight between two successive crosslinking points (M_C_), crosslinking density (Ve), and mesh size (ξ) of an e-beam/gamma crosslinked hydrogel to be used as a wound dressing, the most important role is played by the action of radiation. The Ve is a parameter that describes the number of crosslinking points formed between different polymeric chains (inter-molecular crosslinking) or inside of the same polymeric chain (intra-molecular crosslinking) upon irradiation. The M_C_ is a measure of the degree of hydrogel crosslinking, and ξ is defined as the linear distance between consecutive crosslinking points and the space available between the macromolecular chains [139]. In general, the network parameters depend on many factors, such as composition, nature, and temperature of swelling medium and irradiation dose [140].

Mohamad et al. investigated the biocompatibility of crosslinked hydrogels for wound dressings at different irradiation doses and AA concentrations. They obtained that the Ve significantly increased with the increasing amount of AA in the hydrogel (from 0.41 mol/cm^3^ to 3.06 mol/cm^3^), but the Ve was also influenced by the irradiation dose [50].

Demeter et al. obtained a collagen hydrogel by e-beam crosslinking and demonstrated that Mc depended on the absorbed dose. When the absorbed dose is more than 25 kGy, the radiation effect is almost directly proportional to the G′ modulus and the crosslinking density [51]. In another study, collagen/PVP hydrogels were prepared with the addition of a crosslinking agent. With decreasing crosslinking agent concentration, collagen/PVP systems can be crosslinked via e-beam irradiation with a moderate radiation dose, close to the legally accepted sterilization dose (25 kGy) to obtain the best swelling and optimum rheological properties [119]. Calina et al. demonstrated that the network parameters (M_C_, Ve, and ξ) indicated the formation of a crosslinked structure with a nanostructured mesh size with a typical dimension of 11–67 nm [52].

The sterile and pure TG/PVA/SA wound dressing hydrogels which were prepared through the radiation method showed a value for M_C_ of 77,7939 g/mol, Ve of 2.0 × 10^−6^ mol/cm^3^, and ξ of 38.83 nm [113]. The same authors prepared sterile TG/PVA/PVP hydrogel dressings for simultaneous care of wound infection and wound pain. The TG/PVA/PVP hydrogel dressings showed an Mc value of 43,927 g/mol, Ve of 0.35 × 10^4^ mol/cm^3^, and ξ of 28.05 nm in PBS at 37 °C [111].

The radiation-induced synthesis of novel PHEMA/ITA copolymer hydrogels with different ITA content and the investigation of their potential use in biomedical applications were reported by Tomić et al. They showed that an increase in ITA content gives rise to the enhancement of hydrophilicity. Due to that, polymer–fluid interactions in hydrogels are strengthened, Mc increases, and Ve decreases [81].

### 3.5. Morphology

Scanning electron microscopy (SEM) is used to provide information on sample surface topography and composition. This technique is used to capture the characteristics of the network structure in hydrogels. An important characteristic of hydrogel is the investigation of the structural morphology [141].

The porosity of the dressing matrix is an essential requirement for wound dressing. The porous hydrogel wound dressings are permeable to water vapor and wound exudates. The slow delivery of the drug and the absorption of fluid in the wound depend on the architecture of the hydrogel. The porous structure of polymer films helps absorb wound fluids, keeps the wound dry, and helps supply oxygen to the injury. Cryo-SEM explores the hydrated microstructures of biological specimens. [142].

The TG/PVA/SA hydrogel wound dressing produced by radiation synthesis showed the presence of a porous network, which facilitates the application of the hydrogels in drug delivery and increases the swelling properties of the hydrogels. The hydrogel film had interconnected pores, thus demonstrating crosslinking during synthesis [113]. Cryo-SEM images of the TG/PVA/PVP hydrogel wound dressings depicted the porous surface morphology and crosslinked network structure, which is important for wound fluid absorption and slow drug delivery [111]. In another work, the GA/TG/PVA/PVP hydrogel was incorporated with gentamicin for wound dressing applications. The hydrogel films showed an interconnected, heterogeneous, and highly porous structure, which can lead to a high degree of swelling, permeability, and may support cellular growth [73]. Four years later, they developed a hydrogel dressing to enhance wound healing. The cryo-SEM images of MOG/Carbopol films demonstrated a porous crosslinked network structure. This crosslinked network structure promotes wound healing by enhancing fibroblast adhesion, proliferation, and tissue regeneration [78]. Recently, the cryo-SEM images of TG/SA/PAAM hydrogel in the swollen state showed a highly porous structure in the hydrogel. Furthermore, the porous structure in the hydrogel dressing controls drug loading and drug release. Drug release is dependent on porosity, so by adjusting the pore size, drug release from hydrogels can be controlled. They then showed that the lower pore size led to the slow release of the drug up to a limited period of 9 h as compared to hydrogels with larger pore sizes [112]. The morphology of wound dressing hydrogels was examined containing different concentrations of AA and radiation doses. For this study, the hydrogels exhibited a highly porous mesh network and the pore size of the hydrogels decreased from 61.88 µm (35 kGy dose) to 5.4 µm (50 kGy dose), indicating that the pore size was influenced by AA content as well as e-beam irradiation dose. A smaller pore size is due to the increase in the crosslinking density of the hydrogels at higher AA content and e-beam irradiation dose [50]. Guo et al. showed that the microstructures of PVA hydrogel had a large pore size with uniformity.

After radiation treatment and TA immersion, the pore size was reduced and a dense network was formed, and the lamellar structure disappeared, due to strong hydrogen bonding interactions between TA and CS/PVA leading to the decrease of the pore size [70]. De Silva et al. developed a wound dressing hydrogel using PVP/Carr/PEG by γ-irradiation, and from the SEM analysis, it was observed that the top surfaces of the unirradiated and irradiated hydrogel showed a increased difference. The surface of the unirradiated hydrogel showed cracks in a few places, while the irradiated hydrogel surface did not show cracks [103]. The structure of the collagen hydrogel varied with the irradiation dose, from a more compact structure to a more macroporous structure. It was observed that the crosslinked collagen hydrogel at the dose of 25 kGy has a macroporous state with large pores (50 μm) [51]. The hydrogels have porous network structures and the approximate pore diameter was between 1 and 5 μm [63]. The hydrogels of PVA developed by irradiation followed by freeze-thawing showed a morphology with smaller pores, while those developed by freeze-thawing and freeze-thawing followed by irradiation have a morphology with larger pores. Smaller pores obtained in hydrogels prepared by irradiation and irradiation followed by freeze-thawing allow slower evaporation of water [100]. The HACC/PVA/PEO hydrogel with significant antibacterial activity irradiated at 30 kGy had many micropores, and the pores were uniformly distributed on the surface of the hydrogel. Moreover, it was shown that the pore size of the hydrogels decreased with increasing radiation dose, and the density of the pores on the surface of the hydrogels increased with the increase of the radiation dose [76]. Then in another work, they prepared CS/GEL/PVA hydrogels by the γ-irradiation method for use in wound dressing applications and showed that the microstructure of the hydrogels exhibited many micropores, and the pores were distributed uniformly on the surface of the materials [69]. The PVP/PVA/clay nanocomposite produced by γ-irradiation as a wound dressing showed that the clay particles were finely dispersed through the interconnecting pores of the hydrogel. Moreover, an increase in the clay content leads to a decrease in the pore size [95]. It was shown that the pore size of the PVP/PEG/Agar polymeric hydrogel mainly depends on the radiation dose and the copolymer composition [105]. The XG/PVA/ZnO dressing hydrogels exhibited interconnected porous structures that varied extensively depending on ZnO content. An increased amount of ZnO favors a denser network with a smaller pore size [114]. Zhou et al. found the porous crosslinked network structures with smooth surface morphology in all AgNO_3_/GEL/CM-CS hydrogels. The pore diameter distributions were between 200–600 mm, which were expected to be insufficient for the permeation of nutrients and oxygen from the cells [62]. The PVA/PVP hydrogel has a relatively wider pore structure but a distinguished aggregation of the silver ions is observed as somewhat of a dark particle in the case of PVA/PVP hydrogel-loaded AgNO_3_ [93]. From the microstructure of the pure and PVA/CA/GEL/AgNP composite gels, many micropores were observed on the surface of the materials. The pure and AgNP hydrogels possessed similar micropore structures and showed a tendency to agglomerate with an increase of AgNPs in the gel matrix [85]. The surface of the morphology of the DMAEM/PEO/ZnS nanocomposite at pH 7 (pore size about 5–15 μm) had a relatively uniform pore structure compared to that at pH 4. Moreover, the SEM results revealed that DMAEM/PEO/ZnS nanocomposite exhibited uniform structure and antimicrobial properties [71]. The SEM analysis revealed a PVP/CS/ITA copolymer with a rough surface, full of polymer aggregates with some cavities and hills due to the inhomogeneous effect of radiation during crosslinking processes. After loading the ZnO nanoparticles into the PVP/CS/ITA hydrogel, they obtained a hydrogel with a very rough surface [91]. The PVP/CMC hydrogel produced by irradiation at 30 kGy showed a porous structure and the pore size was 25 µm [35]. Varshney demonstrated that the presence of polysaccharides in aqueous solutions forms a gel network structure that affects the properties of the gel formed upon irradiation. The gels have a highly porous structure and pores of 1–3 µm size can be seen within the porous structure. The well-defined pore structure could be due to the presence of gel-forming polysaccharides and the evaporation of water from them, which form the basic structure before irradiation [82]. The smart PHEMA/ITA copolymer, with ITA content up to 5 mol% was studied upon γ-irradiation. For the PHEMA/ITA gel sample that was not lyophilized, the surface was compact, smooth, and dense, and for the lyophilized PHEMA/ITA gel sample, a heterogeneous distribution of pores in the sample structure was observed. Thus, the authors obtained intelligent hydrogels for the active treatment of skin and wounds [81].

In another article, the authors observed that all the PHEMA/ITA/AgNPs hydrogels with pore size less than 30 nm have relatively good dispersibility. They also proved that AgNPs formed on the surfaces of the hydrogels and were uniformly distributed [64]. The PVP/AgNP hydrogels irradiated at 25 kGy confirmed by SEM analysis that they were well distributed and have a spherical shape in the range of 4–10 nm [65]. Alcântara et al. obtained an antibacterial hydrogel dressing with AgNPs crosslinked by radiation and SEM analysis revealed a uniform distribution of AgNPs on the surface of the hydrogel dressing [83]. The ELT/PVP hydrogel exhibited a porous structure, showing interconnected pores (16 ± 8.42 μm) [72].

### 3.6. Moisture Retention Capability and Water Vapor Transmission Rate

Moisture retention capacity (MRC (%)) is an important factor for wound dressing hydrogels because a moist environment helps to heal the wound faster [143].

The MRC (%) of PVP/PEG/Agar wound dressing hydrogels after 5 h was 60% at room temperature and 40% at 37 °C [105]. The XG/PVA/ZnO nanocomposite retained approximately 50–65% of its water content after exposure to air for 6 h [114]. Experimental investigation showed that hydrogels from CS/PVP/PEG retain after 24 h between 80.52 and 82% humidity [52]. The MRC (%) in 4 h was 69% for the PVP/CMC hydrogels, having a similar moisture retention capacity, regardless of their composition and crosslinking density [35].

An ideal wound dressing must control the water loss from a wound at an optimal rate. The hydrogel prepared as a wound dressing must decelerate the loss of body liquid from the wound and at the same time, maintain a suitable humidity in the wound area. The water vapor permeability of a wound dressing hydrogel should prevent excessive dehydration or edema. An extremely high water vapor transmission rate (WVTR) may lead to wound dehydration, whereas a low WVTR may cause the accumulation of wound exudates. Hence, a dressing with a suitable WVTR is required to provide a moist environment for establishing the best milieu for natural healing [144]. The WVTR values of the hydrogel wound dressings obtained by irradiation are presented in Table 3.

The water vapor transmission rate (WVTR) for normal skin is 204 ± 12 g/m^2^ day, which for injured skin can range from 279 ± 26 g/m^2^ day for a first-degree burn to 5138 ± 202 g/m^2^ day for a granulating wound [145]. Commercial wound dressings demonstrate WVTR values between 76 and 9360 g/m^2^ day [146]. When selecting dressings for wound management, they have to decide which type of dressing can maintain moisture for each type of wound. Therefore, WVTR characterization is important for wound dressing testing.

### 3.7. Drug Release

Wound dressings loaded with drugs can be used as a potential wound dressing with improved healing effects in wound care. The release of the drug-loaded in hydrogels occurs after water penetrates the polymeric networks, and this is followed by diffusion along the aqueous medium. The drug release is related to the swelling characteristics of hydrogels. The release profile of moxifloxacin hydrochloride from the drug-loaded TG/SA/PVA hydrogels showed that the amount of drug released in simulated wound fluid was higher than the release in PBS, pH 2.2 buffer, and distilled water [113]. The release rate of gentamicin from the TG hydrogel was found to be quite fast in both water and simulated wound fluid at 37 °C. The antibiotic release was observed more in water compared to simulated wound fluid [73]. The release profile of the antibiotic drug levofloxacin from the MOG/Carbopol polymer films in pH 2.2 buffer, PBS, and simulated wound fluid was studied. In pH 2.2 the drug release was more pronounced as compared to the other swelling media, thus levofloxacin showed pH-dependent solubility. Moreover, the solubility of the drug was the controlling factor for the release of the drug from the polymer matrix [78]. The drug-loaded TG/SA/PAAM hydrogels in different media indicated that the drug release was higher in PBS and simulated wound fluid as compared to pH 2.2 buffers. They obtained significant release values for lidocaine (34.86%) and amikacin (64.75%) [112].

For the AA/PLST/MMT/CS hydrogels with 10 and 20% of CS, the release of Sulfanilamide reaches equilibrium after 200 min [61]. The hydrogels containing ciprofloxacin hydrochloride (CIP)-loaded PLGA nanoparticles as antibacterial materials using γ-radiation were prepared. After four days, the amounts of CIP released from the PLGA nanoparticles were between 43.38 and 94.63 ± 2.91% [67]. PVA/PVP/CS hydrogels containing antibiotic ciprofloxacin lactate showed that the total amounts of the drug released were 85 and 65% for initial drug contents of 2.0 and 1.0 mg/mL, respectively [97]. The ibuprofen loaded in CS/PVP/PEG hydrogels was investigated at pH = 7.4 and pH = 9.4 (an infected wound). It was observed that about 30–45% of ibuprofen was released in the first 5 h, equivalent to around 30 mg. In the weak alkaline media, the ibuprofen release was lower, reaching 25–35% in the first 5 h [52]. The drug release from DMAEM/PEO/ZnS nanocomposite hydrogel loaded with gentamicin (G), colistin (C), and neomycin (N) at pH 4 and pH 7 was investigated. The neomycin and colistin drugs were more highly released at pH 4 than at pH 7, only the gentamicin release increased from 40% at pH 4 to 96% at pH 7 after 330 min [71]. The amoxicillin drug release on PVP/ITA/CS/ZnO nanocomposite hydrogels was achieved at pH 2.1 and 7.4. The release profile of amoxicillin at pH 2.1 was prolonged and the total amount released after 240 min was 33.5%. At pH 7.4, nearly 67% of the loaded drug was released in the first 60 min [91]. Ciprofloxacin was used as a model antibiotic to study the release properties of the PEDGA/CS hydrogels obtained by γ-irradiation. At 150 min, the cumulative release of PEDGA/ciprofloxacin and PEDGA/CS/ciprofloxacin hydrogels at an irradiation dose of 25 kGy were 87.96 ± 0.83% and 97.34 ± 0.41%, respectively [80]. In vitro cumulative release of sericin from the PVA hydrogels in PBS solution at 37 °C demonstrated that the sericin (50–70%) was released from the hydrogels within 6 h [147]. The TG/PVA/PVP hydrogels were prepared at an irradiation dose of 27.3 kGy. The lidocaine and gentamicin were released from the hydrogel in 360 min and showed different release profiles due to solubility and swelling in different simulated environments. Moreover, hydrogels loaded with these drugs can be useful as dressings in wound care [111]. The PVP/PEO/Agar γ-irradiated hydrogels loaded with neomycin drug showed a neomycin release of about 40% in 48 h [148].

### 3.8. In Vitro Biological Properties

#### 3.8.1. Antimicrobial Studies

Antibacterial hydrogel wound dressings have been developed with the consideration of the fact that moist, warm, and nutritious environments in wound beds provide ideal conditions for microbial growth.

The hydrogels based on CS/PVA/TA presented significant antibacterial properties against *E. coli* and *S. aureus* with an inhibition rate between 95 and 97% [70]. The antimicrobial activity of SSD-alginate hydrogel and SSD-alginate-PG hydrogel produced using γ-irradiation at 2.5 kGy was determined against most encountered bacterial strains in burn wound infection such as *E. coli*, *K. pneumoniae*, *P. aeruginosa*, *A. baumannii*, *E. aerogenes*, and *S. aureus*. The irradiation treatment improved the performance of the formulated hydrogels and the highest inhibiting bacterial growth was observed for SSD-hydrogel loaded with prodigiosin known as an active ingredient with important antifungal, antibacterial, antimalarial, and antineoplastic properties [109]. CPC/PVA hydrogels demonstrated antibacterial activity on the *E. coli* strain, but this property is due to the action of cetylpyridinium chloride [96]. Regarding the antimicrobial effects of gelatin/PVA hydrogel in which various concentrations of AgNP were added, it was demonstrated that a proper concentration of 0.25% AgNP is efficient against *E. coli* and *S. aureus*, as well as MRSA. These values are considered a balance between biological and mechanical properties [63]. The CIP/PLGA-loaded PVA hydrogels denote important antibacterial activity in the *E. coli* and *S. aureus* strains [67]. The hydrogels fabricated from PVA and ws-chitosan showed efficiency only on *E. coli* due to their capability to bind Gram-negative bacteria to the chitosan amino groups [100]. PVP/carrageenan hydrogels incorporated with nanosilver prepared at 25 kGy by in situ reduction of Ag^+^ and hydrogel crosslinking using γ-irradiation showed antimicrobial activity against the common contaminants isolated from burn patients, *P. aeruginosa*, *S. aureus*, *E. coli*, and *C. albicans*. The most effective hydrogels against bacterial strains were those loaded with 100 ppm nanosilver after 3–6 h [102]. PVA/PVP/glycerin/antibacterial hydrogels produced by irradiation at 25 kGy and freeze-thawing were verified for their efficiency against the three bacterial strains *E. coli*, *S. aureus*, and *S. aeruginosa*. In this case, the antibacterial effect generally decreased after irradiation. The hydrogels containing chloramine-T showed an accentuated decrease of antibacterial activity due to its weak resistance at irradiation. Upon irradiation can be generated radiolysis product, which reduces its bactericidal effect. It must be specified that the antibacterial properties of hydrogels containing sulfadiazine sodium salt were not affected by the γ-irradiation [98]. Quaternary ammonium chitosan hydrogel blended with PVA and PEO performed great antibacterial activity against *S. aureus* and *E. coli* [76]. The quaternary ammonium chitosan exhibits important antibacterial activity due to its polycationic structure which is attracted to the surface of bacteria and leads to the alteration of their cell membrane, releasing a significant amount of protein material or cytoplasmatic constituents out from the bacterial cell, finally producing the death of the bacteria [149]. Xanthan-based wound dressing hydrogels containing ZnO nanoparticles crosslinked at 30 kGy were tested to demonstrate the antibacterial activity against *S. aureus*, *E. coli*, and *C. albicans*. The results indicated a broad antimicrobial spectrum, both antifungal and antibacterial, and were correlated with the increase of ZnO nanoparticles included within xanthan dressings [114]. The nanosilver/gelatin/CM-chitosan hydrogels clearly showed antibacterial ability against *E. coli.* The zone of inhibition was wider at 10 mM of nanosilver [62]. The radiation synthesized of 2-(dimethylamino)ethyl methacrylate/PEO/ZnS nanocomposite hydrogel at 20 kGy loaded with antibiotics (colistin, gentamicin, and neomycin) was tested against both bacterial strains (*E. coli*, *P. aeruginosa*, *S. aureus*, and *B. subtilis*) and unicellular fungi (*A. niger*, *A. terreus*, *A. flavus*, and *A. fumigatus*). The presence of ZnS nanoparticles indicates the increase of antimicrobial and antifungal activity on all strains tested, especially those hydrogels loaded with neomycin [71]. PVP/chitosan/itaconic acid/ZnO nanocomposite hydrogel was efficient against *S. aureus* and *E. coli*. The incorporation of amoxicillin into these hydrogels increased significantly the antimicrobial activity [91]. Strong antimicrobial effect and complete inhibition of *P. aeruginosa*, *S. aureus*, and *C. albicans* were observed in the case of PVP/alginate hydrogel dressings containing 70 ppm nanosilver crosslinked by γ-irradiation at 25 kGy [38]. Smart P(HEMA/IA/silver NP) hydrogels were produced in the same step with the reduction of Ag+ by the γ-irradiation method at 12 kGy. The Ag/P(HEMA/IA) hydrogel demonstrated high antibacterial effects on *E. coli*, *S. aureus*, and *C. albicans* with effectiveness even at small silver concentrations [64]. AA/ZnCl_2_ crosslinked hydrogels at 25 kGy indicated that they have good antimicrobial activity against *S. aureus* and *E. coli* strains. The antibacterial effects of AA/ZnCl_2_ hydrogels increased as a function of the ZnCl_2_ concentration [79]. Antimicrobial hydrogel wound dressing was developed, where they included CS dissolved in lactic acid to the original composition of PVP/Agar hydrogel dressing before irradiation. The Gram-negative bacteria *E. coli* growth was not inhibited and the test involving *S. aureus* showed that Gram-positive bacteria growth was hindered in the presence of hydrogel dressings containing CS as compared with the original hydrogel dressing [120]. The synthesized PVP/AgNPs hydrogel showed antimicrobial activity against *P. aeruginosa* and *S. aureus* and the PVA/AgNPs hydrogel indicated that it only had antibacterial activity against *P. aeruginosa* [83]. In another work, the pure PVP hydrogels had no inhibition zone, while the PVP/AgNPs hydrogels (5 mM) presented inhibition zones against all tested bacteria: *B. cereus*, *S. aureus*, *S. epidermidis*, *S. pyogenes*, *A. iwoffii*, *E. coli*, and *P. aeruginosa*. The PVP/AgNPs hydrogels with different concentrations of AgNPs irradiated at 25 kGy were investigated for bactericidal efficacy against *S. aureus*, it was proven that the rate of bacterial reduction increased with the increase of AgNPs content up to 99%, over 6 h (5 mM AgNPs) and 12 h (1 mM AgNPs) [65]. The Psyllium/Carbopol hydrogel dressings with frankincense essential oil developed using γ-irradiation showed efficient antimicrobial activities against *S. aureus*, *E. coli*, and *C. albicans* [150].

The antibacterial activity data for different hydrogel wound dressings are presented in Table 4. The in vitro antimicrobial tests of hydrogels were explored using microorganisms on Gram-positive bacteria (*S. aureus*, *C. albicans*) and Gram-negative bacteria (*P. aeruginosa*, *E. coli*).

#### 3.8.2. Cytotoxicity Studies

It is important to underline that from our study it emerged that the most used polymer for the development of hydrogels by radiation crosslinking for dressing applications is poly(vinyl alcohol). Cytotoxicity studies showed γ-crosslinked PVA hydrogel exhibited partial toxic effects on the L929 fibroblasts by comparison with PVA hydrogels crosslinked with glutaric aldehyde. This behavior has been associated with the partial crosslinking of the PVA polymer and some degradation products resulting after the irradiation process, or it is due to the high radiation dose used for the crosslinking reaction [152].

Considering that wound dressing hydrogels generally have as their main application the healing of skin wounds, this review takes into account the works that refer to the testing of these materials from the point of view of their cytotoxicity on skin-specific cells. The in vitro biological properties of BC/AA hydrogels were assessed by cytotoxicity test on human fibroblast cells. After a 24 h incubation, the hydrogels formulation was found nontoxic and showed cell viability above 88% [50]. The extract of PVA/ws-chitosan/glycerol hydrogels was nontoxic towards L929 mouse fibroblasts; moreover, they showed an accelerated wound healing process [89]. The cytotoxicity test using the NCTC Clone 929 cell line on the PVP hydrogels reinforced with PP showed cell viability above 75%. The percent of cell viability depended on the extract concentration [56]. PVA/alginate hydrogels, after incubation for 48 h, showed a considerable reduction in toxicity on L929 fibroblasts. All hydrogels tested showed a cell viability greater than 85% [84]. The cytotoxicity of AgNP/Gelatin/PVA hydrogels was investigated on normal human dermal fibroblasts showing 80% cell viability. The cell viability was little influenced by the increase in AgNP concentration and the incubation period [63]. The toxicity of CIP/PLGA-loaded PVA hydrogel prepared by γ-irradiation at 25 kGy was tested on human dermal fibroblasts. The studies indicated the non-toxicity of 244 mg CIP/PLGA-loaded PVA hydrogels [67]. In vitro cytotoxicity studies of gelatin/poly (γ-glutamic acid) hydrogels performed on the L929 fibroblasts revealed no cytotoxicity [74]. PVA/ws-chitosan/glycerol hydrogels produced by combining γ-irradiation at 40 kGy with a freeze-thawing cycle demonstrated non-toxicity towards L929 mouse fibroblasts. Moreover, these hydrogels showed cell viability higher than 100% after 24 h of incubation [101]. The ELT/PVA hydrogel demonstrated high cell viability (99.4%) on calcein and ethidium homodimer fibroblasts at lower concentrations (0.10% *w*/*v* ELT), whereas higher concentrations of ELT (0.15–0.25% *w*/*v*) revealed low values of viability [72]. The CM-CS/PEDGA hydrogels did not show significant cytotoxicity on the mouse fibroblast cell line L929, and even an increase in the number of cells was observed at lower extract concentrations (84.3%) [153]. PVA/sericin hydrogels were evaluated to determine the cytotoxicity of an indirect method on L929 mouse fibroblast cells. The cells cultured with the extracts of PVA/sericin hydrogels containing a higher sericin content (25% *w*/*v*) had proliferated more than cells cultured with the extracts of hydrogels containing a lower sericin content [147]. The PVP/AgNPs presented a low toxicity to human fibroblast cell line HaCaT of approx. 10%. For the PVA/AgNPs, cellular death was observed in up to just over 20% of the tested fibroblasts, so the hydrogel could be considered cytotoxic. Therefore, the PVA and PVP hydrogels with AgNPs were shown to be biocompatible, and they were considered nontoxic [83]. The effectiveness of the PVP/AgNPs hydrogels as a wound dressing was investigated for their cytotoxicity using mouse fibroblast cells (L929) and these hydrogels presented cell viability greater than 80% [65]. Cell viabilities for PVA/GEL bilayer hydrogel using 3T3-Swiss albino mouse embryonic fibroblasts were 98% (GEL) and 88% (PVA), respectively, thus showing good biocompatibility [138].

## 4. General Remarks and Future Perspectives

The scientific literature presents hydrogel wound dressings as promising candidates for improving traditional methods in various biomedical applications. Different advanced concepts have been developed in the last twenty years to prepare unique and biocompatible hydrogel wound dressings mainly using ionizing radiation.

This review paper includes methods of characterizing polymer hydrogels crosslinked with ionizing radiation. Furthermore, the paper includes general results published by different research groups that have studied the behavior of hydrogels used as wound dressings.

Each characterization method is essential and helps to obtain a hydrogel with properties that mimic human skin and its healing/regeneration capacity as faithfully as possible. In addition to crosslinking, the irradiation technology ensures dressing sterilization, a fundamental requirement for medical devices that come into contact with skin wounds. Additionally, this review paper includes studies on the antimicrobial and cytotoxicity properties of various hydrogels. Cytotoxicity studies represent an efficient method of testing cell proliferation and are discussed by several authors.

This review paper demonstrates that the hydrogels developed by radiation technologies are excellent dressing materials for wound healing. The crosslinked hydrogels in this way can absorb wound fluids, have good antimicrobial properties, improved functional adhesion, are cost-effective, and are easy to remove.

Hence, the polymers, antibacterial agents, drugs, and radiation synthesis used significantly improved the physicochemical properties of the prepared hydrogel dressings. Since e-beam and γ-irradiation are common sterilization methods, the properties of the irradiated hydrogels at doses of 25–30 kGy were investigated.

In the specialized scientific literature, a small number of clinical studies used hydrogel wound dressings. Therefore, it is necessary to carry out new such studies considering the useful properties of hydrogel dressings obtained by irradiation. The future development of hydrogel wound dressings will lead to much more precise and intelligent properties, biocompatibility, and well-regulated antibacterial activity.

## Figures and Tables

**Table 1:** Classification of preparation methods and irradiation dose.

Hydrogel Composition	Radiation Crosslinking Types	Irradiation Dose	References
Agarose	e-beam	5–30 kGy	[49]
BC/AA	e-beam	35–50 kGy	[50]
Collagen	e-beam	5–100 kGy	[51]
CS/PVP/PEG/AA	e-beam	15–25 kGy	[52]
PEO/PVA	e-beam	20–100 kGy	[53]
PVA/AA	e-beam	10–100 kGy	[54]
PVA/PVP/Carr/silk	e-beam	10–100 kGy	[55]
PVP/PEG/Agar	e-beam	20–50 kGy	[56,57,58]
PVP/PEG/HEMA	e-beam	20 kGy	[59]
PVP/PEG/Starch	e-beam	25 kGy	[60]
AA/PLST/MMT/CS	γ-irradiation	15 kGy	[61]
AgNO_3_/GEL/CM-CS	γ-irradiation	30 kGy	[62]
AgNP/GEL/PVA	γ-irradiation	30–50 kGy	[63]
AgNPs/PHEMA	γ-irradiation	12 kGy	[64]
AgNPs/PVP	γ-irradiation	25–45 kGy	[65]
CHS/SA	γ-irradiation	25 kGy	[66]
CIP/PLGA/PVA	γ-irradiation	25 kGy	[67]
CM-CS/GEL	γ-irradiation	30 kGy	[68]
CS/GEL/PVA	γ-irradiation	40 kGy	[69]
CS/PVA/TA	γ-irradiation	30 kGy	[70]
DMAEM/PEO/ZnS	γ-irradiation	10–30 kGy	[71]
ELT/PVP	γ-irradiation	25 kGy	[72]
GA/TG//PVA/PVP	γ-irradiation	27.3 kGy	[73]
GEL/γ-PGA	γ-irradiation	3–100 kGy	[74]
GEL/PVP	γ-irradiation	5–30 kGy	[75]
HACC/PVA/PEO	γ-irradiation	20–60 kGy	[76]
Lyophilized collagen	γ-irradiation	10–100 kGy	[77]
MOG/Carbopol	γ-irradiation	13.68–41.04 kGy	[78]
PAA/ZnCl_2_	γ-irradiation	25–75 kGy	[79]
PEGDA/CS	γ-irradiation	5–25 kGy	[80]
PHEMA/ITA	γ-irradiation	25 kGy	[81]
PVA/Agar/Carr	γ-irradiation	25–30 kGy	[82]
PVA/AgNPs; PVP/AgNPs	γ-irradiation	25 kGy	[83]
PVA/Alginate	γ-irradiation	25–50 kGy	[84]
PVA/CA/GEL/AgNPs	γ-irradiation	15–25 kGy	[85]
PVA/CM-CS/honey	γ-irradiation	25–40 kGy	[86]
PVA/CS	γ-irradiation	20–50 kGy	[87,88]
PVA/CS/Gly	γ-irradiation	40 kGy	[89]
PVA/Gly	γ-irradiation	25 kGy	[90]
PVP/ITA/CS/ZnO	γ-irradiation	30 kGy	[91]
PVA/PVP	γ-irradiation	5–60 kGy	[92]
PVA/PVP/AgNPs	γ-irradiation	15–40 kGy	[93]
PVA/PVP/aloe vera	γ-irradiation	25–50 kGy	[94]
PVA/PVP/clay	γ-irradiation	10–40 kGy	[95]
PVA/PVP/CPC	γ-irradiation	50 kGy	[96]
PVA/PVP/CS	γ-irradiation	15–70 kGy	[97]
PVA/PVP/Gly	γ-irradiation	25 kGy	[98]
PVA/PVP/Gly/CS	γ-irradiation	25–60 kGy	[99]
PVA/ws-CS	γ-irradiation	30 kGy	[100]
PVA/ws-CS/Gly	γ-irradiation	30–70 kGy	[101]
PVP/Alginate/AgNPs	γ-irradiation	25 kGy	[38]
PVP/Carr/AgNPs	γ-irradiation	25 kGy	[102]
PVP/Carr/PEG	γ-irradiation	25 kGy	[103]
PVP/CMC	γ-irradiation	5–60 kGy	[35]
PVP/k-Carr	γ-irradiation	15–35 kGy	[104]
PVP/PEG	γ-irradiation	25–30 kGy	[105]
PVP/PEG/Agar	γ-irradiation	25 kGy	[106]
PVP/PEG/Alkhydin	γ-irradiation	25–75 kGy	[107]
SF/CS/PVA/AgNPs	γ-irradiation	20–60 kGy	[108]
SSD/SA/PG	γ-irradiation	2.5–120 kGy	[109]
Sterculia gum/PVA/PVP	γ-irradiation	8.42–50.54 kGy	[110]
TG/PVA/PVP	γ-irradiation	9–45.4 kGy	[111]
TG/SA/PAAM	γ-irradiation	34.7 kGy	[112]
TG/SA/PVA	γ-irradiation	9.1–36.3 kGy	[113]
XG/PVA/ZnO	γ-irradiation	10–30 kGy	[114]

**Table 2:** Gel fraction, the swelling degree in water, and PBS of hydrogel wound dressings irradiated between 25–30 kGy.

Hydrogel Composition	Gel Fraction (%)	Swelling Degree (%) in Water	Swelling Degree (%) in PBS	References
Agarose	-	163	-	[49]
BC/AA	-	5704–9903	~3000	[50]
CIP/PLGA/PVA	89–91	-	~250	[67]
Collagen	60	-	260	[51]
Collagen/PVP	84	9000	1450	[119]
GE/PVA/AgNPs	64–78	-	119–231	[63]
GE/IPN/PVP	82–83.4	485–507	-	[75]
GEL/γ-PGA	52.4	~3500	367	[74]
HACC/PVA/PEO	68	3800	-	[76]
PAA/ZnCl_2_	78–84	-	5000–6000	[79]
PEO-PVA	62	1400–2000	-	[53]
PEGDA/CS	95–97		500–600	[80]
PVA/Alginate	80–98	-	700–1000	[84]
PVA/AgNPs	86	240	-	[83]
PVA/CM-CS/honey	55–57	1254–2551	-	[86]
PVA/CS	74–88	50–130		[87]
PVA/Gly	88–93.5	250–350	-	[90]
PVA/PVP	78	42–168	-	[92]
PVA/PVP/AgNPs	86	1400	-	[93]
PVA/PVP/aloe vera	18–35	2600–4600	-	[94]
PVA/PVP/Carr/silk	60–79	-	-	[55]
PVA/PVP/clay	70	210	-	[95]
PVA/PVP/CS	55–90	450–550	-	[97]
PVA/PVP/Gly	36–63	1190–1776	-	[98]
PVA/PVP/Gly/CS	30–52	4500–5400	-	[99]
PVA/ws-CS	61–80	-	-	[100]
PVP/Alginate/AgNPs	77–82	2144–2697	-	[38]
PVP/Agar/CS/LA	80.2–82.8	-	-	[120]
PVP/AgNPs	70–80	2100–3000	-	[65]
PVP/AgNPs	82	110	-	[83]
PVP/Carr/PEG	-	679	-	[103]
PVP/CMC	78–81	81–429	-	[35]
PVP/CS/PEG/AA	87	-	1250	[52]
PVP/ITA/CS/ZnO	54–66	1490–2607	-	[91]
PVP/PEG/Agar	71	1250		[59]
PVP/PEG/Alkhydin	33–71	400–1200	-	[106]
PVP/PEG/HEMA	89	355–385	-	[107]
SSD/SA/PG	-	150	164.5	[56]
XG/PVA/ZnO	83–89	1280–1950	550–892	[114]

**Table 3:** WVTR of hydrogel wound dressings.

Hydrogel	WVTR (g/m^2^ Day)	References
BC/AA	2105–2666	[50]
CS/GEL/PVA	472.32 ± 133.22	[136]
CS/GEL/PVA/PAAM	541.81 ± 54.05	
CS/PVP/PEG	272.67	[52]
GA/TG	188–287	[73]
GEL/PVA/AgNO_3_	4200–4600	[63]
MOG/Carbopol	2461.14 ± 39.16	[78]
PAAM/LAA	106–185	[128]
PVA/CS	40–73.35	[87]
PVA/PVP	89–173.97	[92]
PVP/AgNPs	783.52–940.16	[65]
PVP/Alginate/AgNPs	278.44	[38]
PVP/Carr/AgNO_3_	481–536	[102]
TG/PVA/PVP	582.23 ± 86.55	[111]
TG/SA/PVA	197.39 ± 25.34	[113]
XG/PVA/ZnO	164.89–184.57	[114]

**Table 4:** Antibacterial activity data of the hydrogel wound dressings.

Hydrogel	Antibacterial Agent	Inhibition Zone Diameter (mm)				References
		*S. aureus*	*C. albicans*	*P. aeruginosa*	*E. coli*	
CS/PEG/ZnO	-	-	-	-	20	[151]
CS/PEG/ZnO	GM	-	-	-	30	
DMAEM/PEO/ZnS	CL	15.2	8.5	13.2	12.5	[71]
DMAEM/PEO/ZnS	GM	15.1	8.4	13.1	12.4	
DMAEM/PEO/ZnS	NEO	15.3	8.5	13.2	12.5	
DMAEM/PEO/ZnS	AM	29.5	11.3	21	15.3	
GEL/CM-CS	AgNO3	-	-	-	13.5–19.6	[62]
GEL/PVA	-	15	-	-	15	[63]
GEL/PVA	AgNPs	15–26	-	-	15–27	
PVA/CA/GEL	AgNPs	14–23.2	14.2–17.3	11–23.7	9–24.9	[85]
PVA/CS	TA	10.5 ± 0.3	-	-	10.4 ± 1.1	[70]
PVA/PVP/Gly	AgNO3	19	-	21	17	[98]
PVA/PVP/Gly	SSD	20	-	26	21	
PVA/PVP/Gly	SD-Na	37	-	43	41	
PVA/PVP/Gly	Chloramine T	11	-	11	11	
PVP/Carr	AgNPs	0.8–4.87	-	1.9–9.56	1.2–8.31	[102]
PVP/ITA/CS/ZnO	-	0.4	-	-	0.8	[91]
PVP//ITA/CS/ZnO	AM	2.8	-	-	2.8	
PVP/PEG/Agar	NEO	22 ± 1	-	-	-	[148]
SF/CS/PVA	AgNPs	1.46–1.67	-	1.44–1.59	-	[108]
Alginate	SSD	16 ± 0.3	-	16 ± 0.6	16	[109]
Alginate/PG	SSD	15	16 ± 0.3	16 ± 0.3	16	
XG/PVA	ZnO	25–40	-	-	15–25	[114]

## Data Availability

No new data were created or analyzed in this study. Data sharing is not applicable to this article.

## Abbreviations

AA	Acrylic acid
AM	Amoxicillin
AgNPs	Ag nanoparticles
BC	Bacterial cellulose
CA	Cellulose acetate
CIP	Ciprofloxacin hydrochloride
CHS	Chondroitin sulfate
CMC	Carboxymethyl cellulose
CM-CS	Carboxymethyl-chitosan
CPC	Cetylpyridinium chloride
CS	Chitosan
CL	Colistin
DMAEM	2-(Dimethyl amino)ethyl methacrylate
Dv	virtual dose
Dg	gelation dose
E-beam	electron beam
ELT	Elastin
G	Radiochemical yield
GA	Gum acacia
GEL	Gelatin
Gly	Glycerin
GM	Gentamicin
G(S)	Radiochemical yield of the scission
G(X)	Radiochemical yield of the crosslinking
HACC	Quaternary ammonium chitosan
HEMA	Hydroxyethyl methacrylate
ITA	Itaconic acid
LAA	L-ascorbic acid
MMA	methyl methacrylate
MOG	Moringa oleifera gum
MMT	Montmorillonite clay
MRSA	Methicillin-resistant Staphylococcus aureus
NEO	Neomycin
PAA	Poly(acrylic acid)
PAAM	Poly(acrylamide)
PEGDA	Poly(ethylene glycol)diacrylate
PEG	Poly(ethylene glycol)
PEO	Poly(ethylene oxide)
PG	Prodigiosin
PGA	Polyglutamic acid
PHEMA	Poly(2-hydroxyethyl methacrylate)
PLGA	Poly(lactic-co-glycodic acid)
PLST	Plasticized starch
PVA	Polyvinyl alcohol
PVP	Polyvinylpyrrolidone
SA	Sodium alginate
SF	Silk fibroin
SSD	Silver sulfadiazine
SD-Na	Sulfadiazine sodium
TA	Tannic acid
TG	Tragacanth gum
TS	tensile strength
ws	water soluble
